# 3D Single Molecule Super-Resolution Microscopy of Whole Nuclear Lamina

**DOI:** 10.3389/fchem.2022.863610

**Published:** 2022-04-28

**Authors:** Ashley M. Rozario, Alison Morey, Cade Elliott, Brendan Russ, Donna R. Whelan, Stephen J. Turner, Toby D. M. Bell

**Affiliations:** ^1^ School of Chemistry, Monash University, Clayton, VIC, Australia; ^2^ Department of Microbiology, Monash Biomedicine Discovery Institute, Clayton, VIC, Australia; ^3^ La Trobe Institute for Molecular Science, Bendigo, VIC, Australia

**Keywords:** astigmatism, multiplane imaging, T cell, nuclear envelope, convex hull

## Abstract

Single molecule (SM) super-resolution microscopies bypass the diffraction limit of conventional optical techniques and provide excellent spatial resolutions in the tens of nanometers without overly complex microscope hardware. SM imaging using optical astigmatism is an efficient strategy for visualizing subcellular features in 3D with a *z*-range of up to ∼1 µm per acquisition. This approach however, places high demands on fluorophore brightness and photoswitching resilience meaning that imaging entire cell volumes in 3D using SM super-resolution remains challenging. Here we employ SM astigmatism together with multiplane acquisition to visualize the whole nuclear lamina of COS-7 and T cells in 3D. Nuclear lamina provides structural support to the nuclear envelope and participates in vital nuclear functions including internuclear transport, chromatin organization and gene regulation. Its position at the periphery of the nucleus provides a visible reference of the nuclear boundary and can be used to quantify the spatial distribution of intranuclear components such as histone modifications and transcription factors. We found Alexa Fluor 647, a popular photoswitchable fluorophore, remained viable for over an hour of continuous high laser power exposure, and provided sufficient brightness detectable up to 8 µm deep into a cell, allowing us to capture the entire nuclear lamina in 3D. Our approach provides sufficient super-resolution detail of nuclear lamina morphology to enable quantification of overall nuclear dimensions and local membrane features.

## Introduction

Fluorescence microscopy techniques are routinely employed in biological research, making use of a variety of fluorophore labelling strategies to enable specific visualization of cellular structures and dynamics. The nuclear lamina is a continuous protein meshwork comprised of lamins, such as lamin A/C and B1, and is located within the inner membrane of the nuclear envelope. Lamins are key structural proteins that contribute to the structural integrity of the nucleus and provide a scaffold in which chromatin domains can be anchored. In addition, nuclear lamina has been shown to have roles in chromatin organization, gene regulation and transcription (Burke and Stewart 2013). Mutations in the genes encoding lamin proteins disrupt the integrity of the nuclear lamina resulting in structural aberrations, dysregulated gene expression and genomic instability, and have been associated with diseases collectively termed laminopathies (Rankin and Ellard 2006). Additionally, the nuclear lamina is a recognizable boundary of the nucleus that when imaged, can be used as a reference structure for describing the spatial arrangement of intranuclear components including histone modifications and transcription factors. Of particular interest, imaging the nuclear lamina could aid in characterizing the nuclear interior of immune cells where the 3D spatial organization of the genome is associated with immune cell function i.e., activation, differentiation, and cell-type specific function (Pongubala and Murre 2021). Direct observation of specific nuclear features, however, can be challenging. This is partly due to the compactness of the nuclear space, but also because conventional optical methods are diffraction limited to ∼200 nm imaging resolution at best, insufficient for resolving biomolecular and subcellular detail sized in the tens of nanometers.

Super-resolution fluorescence microscopies (SRFMs) are ideal for visualizing nuclear features (Miriklis et al., 2021), providing target specificity, subdiffraction resolution and capability for 3D imaging (Schermelleh et al., 2019). SRFMs include the deterministic methods such as Stimulated Emission Depletion (STED) (Klar and Hell 1999; Klar et al., 2000) and Structured Illumination Microscopy (SIM) (Gustafsson 2000) that optically engineer the excitation source to yield subdiffraction output. SRFMs also include the stochastic methods that harness the photoswitching properties of fluorophores to achieve single molecule localization microscopies (SMLMs) (Betzig et al., 2006; Hess et al., 2006; Rust et al., 2006; Sharonov and Hochstrasser 2006) including direct stochastic optical reconstruction microscopy (*d*STORM) (Heilemann et al., 2008). SMLMs offer outstanding imaging gains—as good as 20 nm resolution—and make use of simple organic dye molecules as labels. SMLMs are performed by fitting diffraction-limited intensity profiles of individual SM emissions i.e., the point-spread function (PSFs) and deducing its spatial coordinates. Mapping several hundred thousand (up to a few million) SM coordinates yields the super-resolved image: a pointillistic reconstruction of the fluorophore-labelled structure.

2D-SMLMs can be upgraded for 3D imaging by optically engineering typically 2D symmetrical PSFs to be encoded with axial information. The astigmatism method (Huang et al., 2008a) uses a single cylindrical lens in the imaging path to laterally distort PSFs in *x* and *y* based on the *z* position of the emitter*.* Decoding these distorted PSFs during the localization procedure yields SM coordinates in all 3 spatial dimensions. Astigmatism provides ∼1 µm *z*-range, sufficient for capturing some cellular components such as individual cytoskeletal filaments (Xu et al., 2013) and subnucleolar bodies (Rawlinson et al., 2018) in 3D. Visualizing larger structures or across larger volumes, however, requires additional strategies to improve the imaging depth. One strategy is to stack multiple 1 µm *z*-ranges by scanning the microscope objective through the whole cell, capturing SM emissions throughout. This was first demonstrated as a follow up of PSF astigmatism, employing a dual fluorophore system (activator and reporter dye for STORM) to capture multiple focal planes of mitochondria and microtubules up to a depth of 3 µm (Huang et al., 2008b). A purpose-built multifocus microscope has also been reported to enable large depth 3D SMLM by introducing a focal-splitting prism to the imaging path for simultaneous acquisition of nine focal planes (Hajj et al., 2014). Alternatively, adaptive optics (Shechtman et al., 2014; Shechtman et al., 2015; Mlodzianoski et al., 2018) or emission phase-modulating masks (for double-helix PSFs) (Pavani et al., 2009; Carr et al., 2017) can engineer PSFs with more complex distortions for increased imaging depths up to 6 µm without objective lens scanning. These latter strategies, however, utilize expensive optical hardware that require specialized expertise to install, align and calibrate. Furthermore, not all PSF engineering strategies are immediately compatible with the acquisition prerequisites for SMLM: bright emitting fluorophores and well resolved SM emission patterns. For this work, we demonstrate 3D *d*STORM of whole nuclei using hardware already available, a motorized objective turret on a home-built SM super-resolution microscope (Whelan and Bell 2015a) and a single cylindrical lens placed in the emission path.

Our method for whole nuclei 3D SMLM employs the stepwise acquisition of multiple imaging planes manipulated with astigmatism and the most popular fluorophore for *d*STORM, Alexa Fluor 647 (Alx647), an organic dye that exhibits ideal photoswitching properties for *d*STORM when subjected to a high laser flux and reducing thiol buffer [100 mM mercaptoethylamine (MEA)]. We harnessed the performance of Alx647 for whole nuclei 3D *d*STORM, maintaining bright photoswitching for over an hour that was detectable up to 8 µm deep in a fixed cell. With such prolonged laser exposure throughout the entire labelled sample, a reasonable assumption is that some fluorophores would photobleach before being detected, resulting in lower fluorophore density and poorer image quality of the structure. A previous report to capture whole nuclear lamina in 3D employed a continuous re-labelling strategy of Alx647 throughout the SM acquisition to maintain sufficiently high fluorophore density (Venkataramani et al., 2018). We found conventional immunolabelling provided sufficient fluorophore density throughout the entire depth of the cell, even after an hour of continuous exposure to high laser power. With no signs of extensive photobleaching, whole nuclear lamina could be imaged comfortably, resulting in super-resolution detail throughout the structure.

Here we utilize the advantageous performance of Alx647 for whole nuclei 3D *d*STORM of lamin B1 in primary mouse T lymphocytes (T cells) and cultured COS-7 cells, as well as quantification of nuclear volume and surface areas using convex hull fitting. The fit also provides a 3D reference boundary of the nucleus, useful for mapping spatial distributions of intranuclear components. Interrogation of the 3D model also revealed sub-structures within the nuclear lamina membrane, including folds, blebs and cone-like invaginations potentially indicative of nuclear transport channels. Our assay for 3D whole nuclei super-resolution imaging reveals both the global microscopic perspective of the nuclear membrane as well as local nanoscopic features found therein.

## Materials and Methods

### COS-7 Cell Culture

COS-7 cells (African green monkey kidney fibroblast-like, ATCC CRL1651) were cultured in Dulbecco’s Modified Eagles Medium (DMEM—high glucose) supplemented with 10% fetal bovine serum and 1% penicillin-streptomycin, and incubated at 37°C, 5% CO_2_. For imaging, cells were seeded onto high precision coverglass slides (Marienfeld 18 mm diameter #1.5H) and allowed to reach ∼70% confluency before fixation and immunolabelling.

### Isolation of Naive CD8^+^ T Cells

Female C57BL/6 mice of 6–8 weeks of age were housed in specific-pathogen free conditions at the Monash Animal Research Platform. Experimental procedures were conducted in agreement with the guidelines specified by the Monash Animal Ethics Committee. Spleens were extracted from female C57BL/6 mice and prepared for single cell suspensions. For cell sorting, single cell suspensions were resuspended in PBS and stained with anti-CD8α-fluorescein isothiocyanate (BD Pharmingen), anti-CD44^−^ phycoerythrin cyanine 7 (TONBO biosciences), anti-CD62L-allophycocyanin (Biolegend) and LIVE/DEAD™ Fixable Aqua Dead Cell Stain (Thermo Fisher Scientific). Naive CD8^+^ T cells (CD44^lo^, CD62L^hi^) were sorted using a BD Influx™ Cell Sorter and adhered to Nunc™ Lab-Tek™ II Chambered Coverglass (Thermo Scientific) using Cell-Tak™ Cell and Tissue Adhesive (Corning) before fixations and immunolabelling.

### Fixation and Immunolabelling

COS-7 and naive CD8^+^ T cells were fixed with 3.7% formaldehyde in PBS for 10 min at 37°C in a humid incubator, then washed twice with PBS followed by permeabilization with 0.1% Triton X-100 in PBS for 10 min at R.T. Fixed cells were blocked in 5% bovine serum albumin in PBS for 30 min at R.T., and immunolabelled first with rabbit anti-lamin B1 primary antibodies (1:1,000 dilution, Abcam) then anti-rabbit secondary antibodies conjugated to Alexa Fluor 647 (1:200 dilution, Thermo). Labelled cells were post-fixed with 3.7% formaldehyde in PBS for 5 min at R.T. before storage in 0.05% sodium azide in PBS at 4°C. Cells were images within 1 week of labelling.

### Microscope Setup

SM imaging was performed on a home-built super-resolution microscope, as previously described (Whelan et al., 2014; Whelan and Bell 2015b; Rozario et al., 2020), based around an Olympus IX81 inverted fluorescence microscope frame fitted with a TIRF ×100 1.49 NA oil objective, Oxxius 638-nm laser diode and Andor iXon EM-CCD detector. Micromanager (Edelstein et al., 2010) was used to set acquisition parameters. To induce SM astigmatism for 3D imaging, a cylindrical lens (f = 1,000 mm) was placed in the emission path just after the exit port of the microscope frame (Supplementary Figure S1).

### Astigmatism Calibration

Calibration for astigmatism was based on the original report of 3D SM astigmatic imaging (Huang et al., 2008a). The calibration sample comprised 100-nm Tetraspeck fluorescent beads (Thermo T7279, 1:10,000 dilution) suspended in a glycerol-based water soluble jelly (refractive index, RI ∼1.46) diluted into water to yield a final RI of ∼1.35–1.40. The suspension was added to a Nunc™ Lab-Tek™ II Chambered Coverglass chamber and allowed to air dry overnight at 45°C before imaging. With the cylindrical lens (f = 1,000 mm) placed in the imaging path, a Tetraspeck was scanned through 4 µm in *z* with 10-nm intervals and acquired for 100 ms at each step. Frames were processed through rapi*d*STORM (Wolter et al., 2012) to determine the PSF width in *x* and *y* at each *z*-step, by selecting “Store PSF width.” Similar to 2D *d*STORM, the 2D Gaussian fitted onto each PSF provided the sigma value (width) in *x* and *y* used for the calibration. The data was cropped to a 1 µm *z* range such that the mid-point was where PSF width in *x* was nearest to PSF width in *y*, i.e., the ideal *z* imaging plane of objective. The calibration was smoothed with a 10-point B-spline function and exported from the rapi*d*STORM output “3D PSF width calibration table.” This calibration file was modified by increasing the *z*-coordinate value by increments of 400 nm to match the movement of the objective for each *z*-plane imaged using astigmatic 3D *d*STORM.

### Whole Nuclei Imaging

**FIGURE 1:**
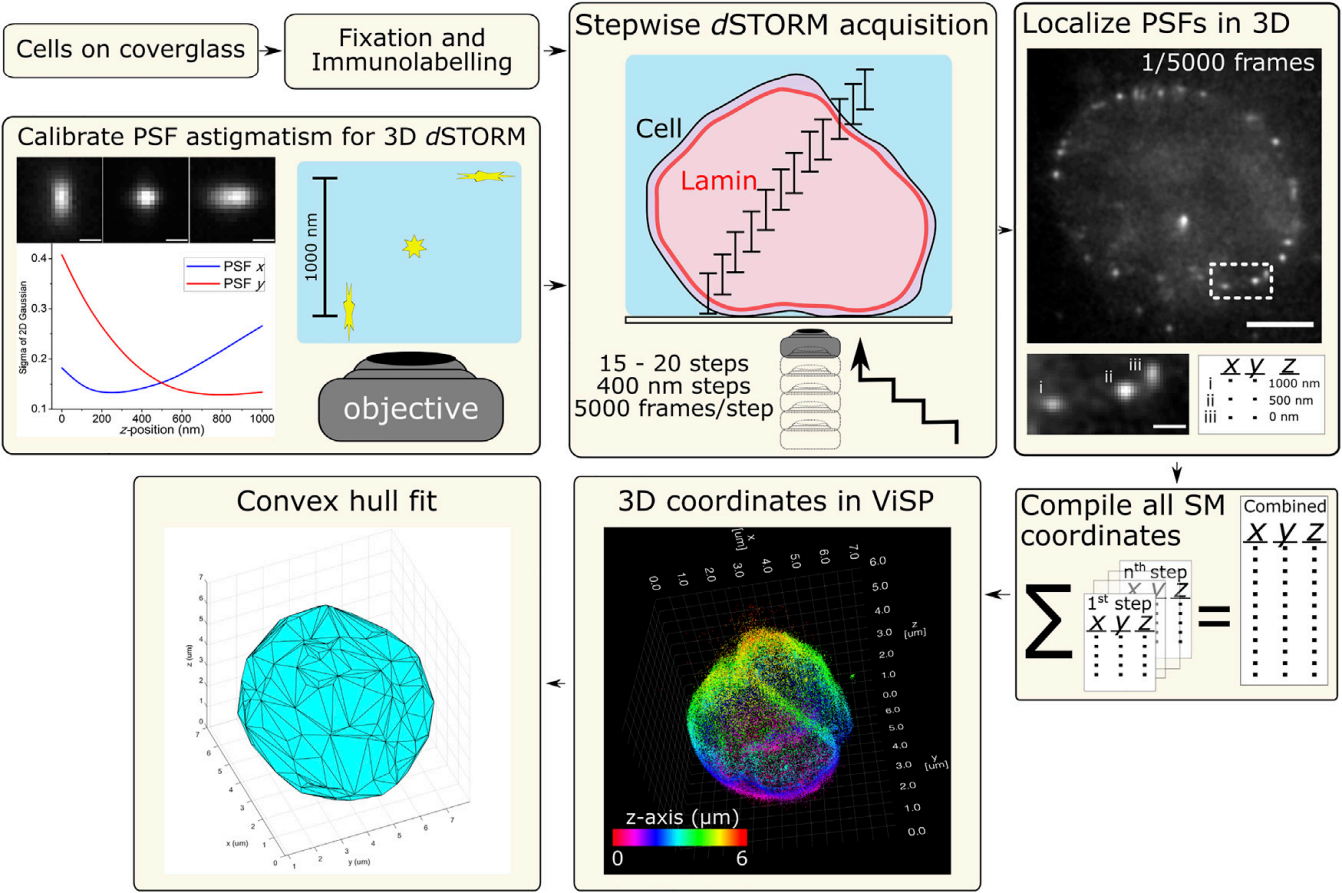
Schematic workflow for 3D *d*STORM of whole nuclear lamina. Procedures for cell sample preparation and imaging are detailed in the *Materials and Methods* section. Cells were fixed and immunolabelled for lamin B1 with the photoswitchable fluorophore Alexa Fluor 647 (Alx647). The microscope setup was adapted for 3D *d*STORM (optical astigmatism) using a cylindrical lens (f = 1,000 mm) in the imaging path to distort the point-spread-function (PSF) of point emitters, here calibrated using a 100-nm fluorescent bead elongated in the lateral direction (*x* and *y*) as a function of axial position (*z*). With labelled cell samples, SM photoswitching was induced and multiple *z*-slices of nuclei were imaged with each slice capturing 1 µm in *z*. Imaging was performed from the bottom with the objective being raised in 400-nm steps, resulting in the overlapping of axial slices and requiring between 15–20 steps to reach the top of the cell. At each step, SM emissions were acquired (5,000 frames at 50 Hz) and localized in 3D using rapi*d*STORM with 3D calibration. Scale bars of raw data = 5 µm (full frame) and 1 µm (zoom frame). All SM coordinates from each step are compiled into a text file. ViSP can be used to visualize the SM coordinates in 3D, revealing the imaged structure. SM coordinates can be color coded either for height (relative position in *z*) or density based on the number of neighboring SM coordinates. The coordinates lists were also used for fitting by convex hulls to quantify nuclear lamina surface area and volume.

Cells were imaged in a switching buffer of 100 mM mercaptoethylamine (MEA) at pH 8.5 (adjusted with 1 M potassium hydroxide) in PBS. The laser was used at full power (150 mW resulting in ∼3 kW/cm^2^) to induce Alexa Fluor 647 SM photoswitching, observed as “blinking” during acquisition. Cells were imaged from the bottom up where, at each step, 5,000 frames of photoswitching SM emissions were acquired at 50 Hz (20 ms exposure), then the objective was shifted up *z* by 400 nm to the next step. Typically 15–20 steps were required to cover the entire height of the nuclear lamina signal. The raw data from each step was processed for 3D SM localization in rapi*d*STORM using the appropriate calibration file that matched the step’s relative position through the cell, e.g., 1st step *z*-range: 0–1,000 nm, 2nd step *z*-range: 400–1,400 nm, etc. The resulting SM coordinate list from each step was compiled into a single text file that was imported into ViSP (Beheiry and Dahan 2013) for visualizing nuclear lamina structure and enabling colour coding each SM coordinate for its position in *z* (Figure 1).

### Convex Hull Fitting

SM coordinate lists from all *z*-slices were combined for each cell and imported into a custom written MATLAB 2020a script that processed the data through a DBSCAN (Ester et al., 1996)-based clustering function to identify individual nuclei based on coordinate density. Convex hull fitting was applied to determine the outer boundaries of each of these dense regions i.e., the nuclei boundaries as imaged with lamin B1. Hulls comprised the outermost coordinates of these dense regions and connected as triangular faces that wrapped around each nuclear boundary. For this work, the sum of these faces was used as the nuclear surface area, and the volume within the fitted hull was determined as nuclear volume.

## Results

### Multiplane Astigmatic PSF Imaging for Whole Cell Super-Resolution

Conventional widefield imaging with *z*-scanning was capable of imaging whole cell nuclear lamina in 3D. As expected, the images showed poor contrast of the nuclear boundary and lacked structural detail in the membrane. A side-on projection showed severely reduced fluorescence signal at the top of the cell compared to the bottom (Supplementary Figure S2), indicative of a reduced efficiency for imaging deeper into biological samples.

Application of *d*STORM improved the resolution of the nuclear lamina (Supplementary Figure S3), revealing a more distinct boundary of the nucleus compared with diffraction-limited widefield imaging and enabled measurements of T cell nuclear lamina thickness within the subdiffraction range (96 nm, 81 and 160 nm); a ∼3-fold improvement over diffraction-limited measurements of the same lamina sections (361 nm, 428 and 380 nm). Our *d*STORM derived values for T cell nuclear lamina are comparable with those from previous SMLM measurements of nuclear lamina of U2OS cells: ∼200 nm (Venkataramani et al., 2018) and MEF cells: ∼100 nm (Nmezi et al., 2019).

For 3D *d*STORM, astigmatism was induced using a cylindrical lens that distorted PSFs in *x* and *y* based on each emitting molecule’s *z* position above or below the objective imaging plane, i.e., where the PSF is in focus. 100-nm fluorescent beads (Tetraspecks) were used to calibrate PSF distortion as a function of *z* position by plotting the relative axial position from the focal plane of an emitting bead against the lateral elongation of its PSF (width in *x* and *y*). With this 3D calibration (as described in *Materials and Methods*), the 3D coordinates of SM emitters in labelled nuclei could be determined within a 1 µm *z* range without the need for scanning in *z* (Figure 1).

The usable *z* range for this astigmatism approach is limited by the loss of integrity of distorted PSFs beyond approximately ±500 nm in *z* from the focal plane. Beyond this ∼1 µm *z* range, a defocused PSF from a single emitter becomes too challenging to distinguish from noise and may appear to have multiple lobes of maximum signal that, when rendered for *d*STORM, would be imprecisely localized as multiple SM coordinates. Another issue with lateral distortions beyond ±500 nm in *z* is that signal intensity from the emitter is spread across more pixels, reducing the likelihood of that PSF reaching the threshold for being selected for localization. While not affecting a calibration Tetraspeck bead that contains multiple fluorophores, a single fluorophore with a finite photon budget would become increasingly difficult to localize when its PSF is excessively elongated. To increase the achievable *z* range for whole cell imaging, we captured multiple *z* planes affected by astigmatism, each providing 1 µm in *z*, stacked in a overlapping fashion accommodate the localization of less distinguished PSF elongations at either extreme of the range (+500 and −500 nm).

For *d*STORM acquisition, a switching buffer was added to the fixed and labelled cells, and the laser was turned to full power to induce Alx647 photoswitching. In order to penetrate the entire cell depth, the laser was aligned straight up (epifluorescence mode) and multiplane imaging was performed from the bottom up. This is different to inclined laser excitation modes [HILO (Tokunaga et al., 2008) or quasi-TIRF (Whelan et al., 2014)] typically used to improve SM photoswitching for 2D *d*STORM. We found Alx647 exhibited sufficient photoswitching properties up to 8 µm in *z* when induced with epifluorescence laser illumination (Supplementary Figure S4). The brightness and frequency of Alx647 photoswitching (blinking) observed during acquisition was a direct visual indication for the eventual quality of the rendered *d*STORM image. Individual frames of raw acquired data showed well resolved PSFs with a noticeable range of astigmatism lengths, enabling 3D localization using rapi*d*STORM with 3D calibration (Supplementary Figure S4). Remarkably, Alx647 maintained desirable brightness and photoswitching frequency throughout acquisition of the whole cell depth—a total duration of ∼1 h with continuous high laser power exposure—to cover a total *z* range of up to 8 µm.

To capture the entire depth of nuclear lamina using 3D *d*STORM, we employed a stepwise acquisition strategy together with SM astigmatism (Figure 1). Each step of acquisition provided a 1 µm *z* range of 3D data—effectively a 1 µm *z* slice through the labelled nucleus. Following acquisition of each slice, the objective was raised by 400 nm so slices overlapped, allowing SM emitters to be localized more frequently throughout the sample. This is especially important for emitters at the extreme edges of the 1 µm *z* range that are not collected as efficiently as the in-plane emitters due to the spreading of photons across more elongated PSFs. The same 3D calibration file for 1 µm in *z* was applied for all *z* slices where only the final *z* coordinates were offset to match that slice’s position in the sample, i.e., the *z* coordinates were offset incrementally by +400 nm to match the distance between each slice.

By inspecting rendered 3D segments from each imaged *z* slice, we can appreciate the spatial detail contained within a whole 3D nucleus model (Supplementary Figure S4). The bottom of the nucleus (*z* = 1 µm) shows a continuous layer of lamin B1, with some regions higher in *z* most notably at the edges where the nuclear lamina curves upward. At the middle of the nucleus (*z* = 3 µm) a distinct ring is observed with very few spurious localizations within the ring, indicative of the high labelling density and specificity of the lamin B1 antibodies. The ring, while revealing its axial position in *z* (by colour) also shows a steady lateral shift outward from its center highlighting curvature along the *z* plane. At the top of the nucleus (*z* = 7 µm), a much smaller area of lamin B1 is observed where the center portion is slightly higher than the edges, effectively the cap of the nuclear membrane. This also demonstrates that out-of-focus emission between the objective and the desired focal plane 7 µm deep do not interfere with 3D SM imaging at these depths.

Applying multiplane 3D *d*STORM allowed visualization of whole nuclear lamina using the combined coordinate data. Upon inspection of the 3D image, ripples were observed when viewed from the side-on (Supplementary Figure S5). Scrutiny of the data from each slice revealed that the majority of localizations (59%) arose from emissions within the center third of the 1 µm *z* slice (*z* ∼ 330–660 nm). When combined with all other slices, each majority region formed an intensity peak that resulted in a rippling through the height of the nucleus. We found that rippling comes from the stacking of multiple astigmatic *z* slices and the spatial frequency of rippling could be modulated by varying the *z* increments during acquisition (100 nm, 400 and 1,000 nm). Smaller *z* increments increased the amount of overlap thereby reducing the space between the center regions of *z* slices and consequently the differences in intensity through *z*, resulting in a smoother 3D model (Supplementary Figure S6).

Lateral drift of the sample was minimal despite ∼20 separate motions of the objective for *z* scanning during acquisition. Viewing all imaged *z* planes from the bottom up (*xy* view, Supplementary Movie S1), the zenith of nuclei were found to be positioned reasonably well in *xy* based on the middle and lower *z* planes. Allowing samples to settle on the oiled objective for at least 10 min prior to acquisition was crucial in minimizing drift.

Step size for multiplane 3D *d*STORM constitutes an important imaging parameter to consider alongside fluorophore choice, exposure time, frames acquired per slice and total duration of acquisition per cell. While using the smallest possible step size (100 nm) would be ideal for the smoothest possible 3D model, the increased objective movement and number of slices increases the chances for drift caused by hardware errors in *z*. Any error associated with the absolute *z* value of the motorized microscope objective turret (even if only a few tens of nanometers) together with the *z* localization precision of PSFs for 3D *d*STORM (∼50 nm) would steadily worsen with each step through the height of the cell. This would lead to additional false SM coordinates occurring in *z* due to an apparent smearing of a single emitter as it is mis-localized across multiple *z* slices. For this work, we settled on using 400 nm steps to minimize the objective movement and total acquisition time required to capture whole nuclei, and despite incurring some rippling in the final model, we determined this would minimize overall localization artefacts.

Between 20–30 overlapping *z* slices (400 nm steps, 5,000 frames/step) were sufficient to capture the entire heights of COS-7 and T cell nuclei. Mouse T cells adhered onto microscope coverglass using Cell-Tak showed nuclei that were relatively spheroidal having similar width and heights of ∼5–6 µm. COS-7 cells cultured directly onto the coverglass showed nuclei that were ∼30 µm laterally but with heights only up to ∼8 µm.

### Measuring Area and Volume of Whole Nuclei

Whole nuclei were rendered in ViSP (Beheiry and Dahan 2013), a program built to visualize and process 3D SMLM output data. By assigning SM coordinates a colour based on *z*-position, for example, the entire volume and hollow nature of the nuclear lamina can be readily observed (Figure 1). Rendering a relatively continuous 3D structure with few spurious SM localizations highlights the efficiency of immunolabelling lamin B1 to reveal a complete nuclear boundary when imaged. As such, the nuclear lamina can serve as a distinct demarcation of the nuclear envelope and thereby nuclear space and volume when quantifying the spatial distributions of intranuclear components.

**FIGURE 2:**
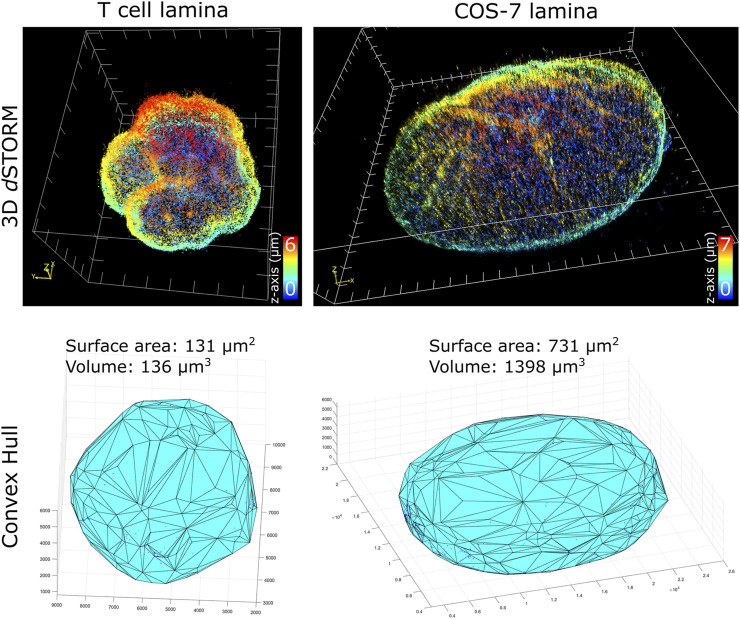
Quantification of nuclei dimensions using 3D *d*STORM of lamin B1 and convex hull fitting. (top row) Whole nucleus of T cell and COS-7 cell visualized in ViSP using SM coordinates acquired from 3D *d*STORM up to 6 µm in *z*. (bottom row) 3D convex hull fitting using SM coordinates to determine approximate surface area and volume of whole nuclei.

To characterize nuclei dimensions, we first used the DBSCAN algorithm (Ester et al., 1996) to identify individual nuclei based on overall SM density, then connected the outermost SM coordinates to form triangular faces that culminate into the 3D convex hull encapsulating the boundary of each nucleus. Transforming individual coordinates into a continuous structure allowed the surface area, as well as the volume within the enclosed hull, to be calculated. Comparing the cell types imaged (Figure 2), unsurprisingly, COS-7 nuclei were much larger with more nuclear surface area than T cell nuclei. In the same imaging field of view (25 µm by 25 µm), we could capture either a single COS-7 cell or multiple (up to 6) T cells within the same imaging period (∼1 h), mitigating the time required to capture T cells compared to COS-7 cells. The convex hull fitting was useful to segregate individual nuclei and subsequently measure the dimensions of each T cell nucleus. The multi cell imaging and analysis demonstrates a viable strategy to increase the throughput of quantitative super-resolution imaging of T cell nuclei.

Of the T cell nuclei imaged (*n* = 8 cells, avg. volume ±SD = 127 ± 15 μm^3^, avg. surface area ±SD = 127 ± 10 μm^2^), interestingly, the surface area to volume ratio (SA:V) of each nucleus was close to 1. Perfect spheres have a SA:V of 3/radius, and since the approximate radius of each T cell nuclei was ∼3 μm, this shows empirically that our sample preparation and imaging assay results in nuclei being closely spherical. This indicates that our protocol for adhering T cells to the coverglass substrate using Cell-Tak and centrifugal force induced little to no compression of the cells. Determining the sphericity of nuclear lamina could be a useful parameter for characterizing lamin-related diseases where nuclear envelope structure or nuclear shape is affected. Additionally, since SA:V is used as a metric for bacterial growth (Ojkic et al., 2019; Shi et al., 2021), there may be potential application for using whole cell 3D *d*STORM to study T cell metabolism based on measured SA:V.

Nuclei membrane substructures such as membrane folds and blebs being more concave were excluded from the convex hull fit and thus require more sophisticated fitting procedures to be mapped autonomously. In this work, we measured these membrane features individually based on the 3D nuclei models rendered in ViSP.

### Membrane Features of Nuclear Lamina in COS-7 Cells and T Cells

**FIGURE 3:**
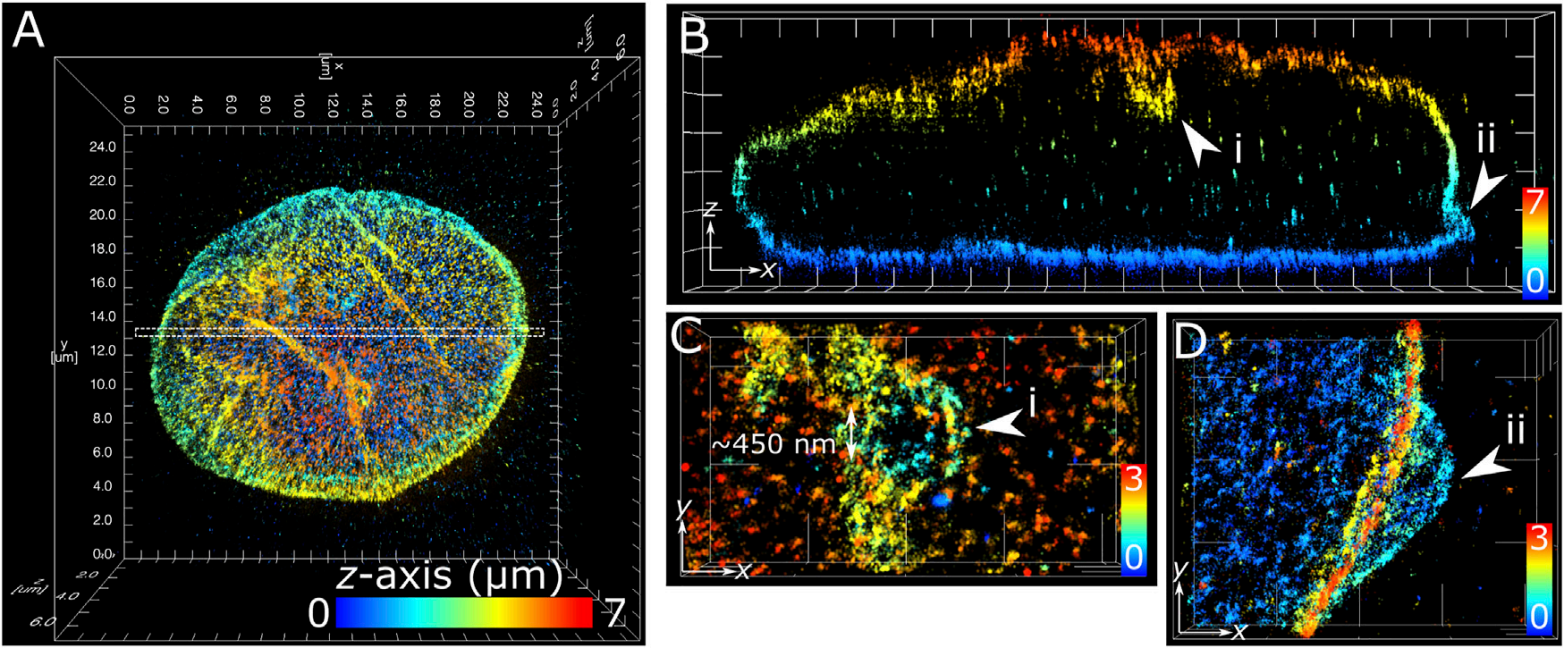
Subdiffraction resolution of membrane features in COS-7 nuclear lamina. Fixed COS-7 cell labelled for lamin B1 with Alexa Fluor 647 and imaged using multiplane astigmatic 3D *d*STORM, 24 µm by 24 µm in *xy* and up to 7 µm in *z*. SM coordinates were rendered in ViSP and color-coded for height. Color scale in each panel indicates *z*-axis range. **(A)** Whole nuclei visualized from the top down *xy* 2D projection. **(B)** A 300 nm section in *xy* from the dotted region in **(A)** visualized from the side reveals sub-lamina features including (i) indentation at the top of nucleus and (ii) outward protrusion at the bottom of nucleus. **(C)** Magnified feature from (i) viewed from the top down reveals a circular indent of outer width ∼1 µm and inner width ∼450 nm. **(D)** Magnified feature from (ii) viewed from the top down reveals an outward swell ∼2 µm wide at the base of the nuclear lamina. Grid ticks in all images = 1 µm.

**FIGURE 4:**
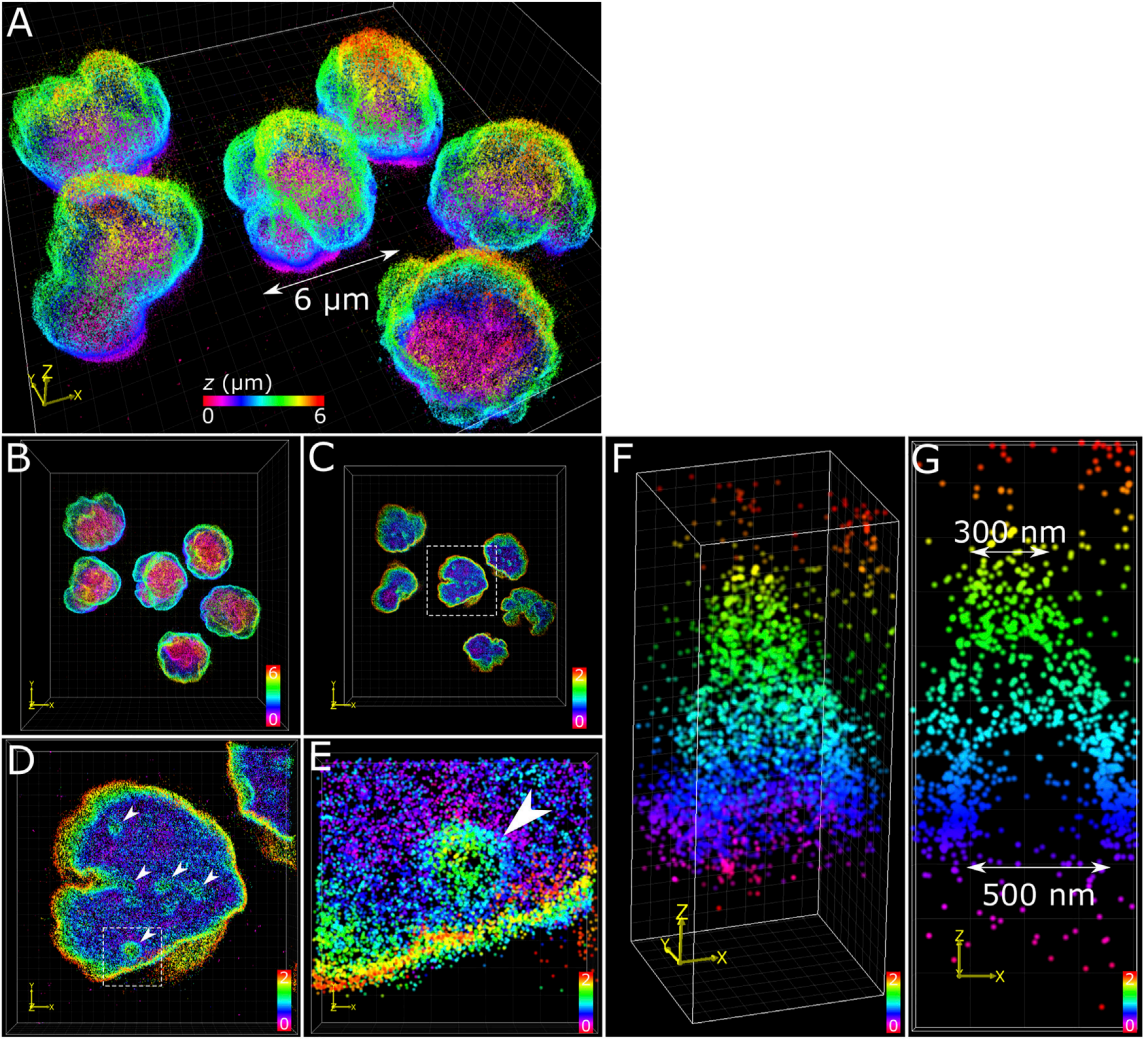
T cell nuclear invaginations visualized with 3D *d*STORM. **(A)** Fixed mouse T cells labelled for lamin B1 with Alexa Fluor 647 and imaged using 3D *d*STORM. **(B)** Top-down projection of the entire height (6 µm) of T cells from a. **(C)** The bottom 2 µm of T cells with the same color gradient used. **(D)** Zoom in on a single nucleus (from white dotted box in c) showing distinct height protrusions, indicated by white arrows. **(E)** A single protrusion (from white dotted box in d). **(F)** 3D visualization of the feature isolated from e reveals a cone-like structure. **(G)** Cross-section through the feature in *y* shows the bottom portion to be relatively void of lamin B1 signal, suggesting a hollow structure. The feature measured 500 nm at the base and 300 nm at the top (toward the nuclear interior). Color bars in each panel indicate *z*-positions of SM coordinates in µm.

The application of multiplane 3D *d*STORM provided excellent subdiffraction resolution along with the whole-cell spatial context of the target protein, demonstrated here with lamin B1 revealing membrane-like features in cultured COS-7 cells and surgically extracted mouse T cells. Some common features include shallow inward folding of the lamina (<1 µm), deep folds into the nucleus reaching up to several µm, and outward blebbing at local regions along the lamina (Figures 3, 4). Also observed along folds were ring-like features that extended into the nucleus of COS-7 cells (Figure 3C) and T cells (Figure 4E). While these features were sized just at or above the diffraction limit (∼200 nm), performing *d*STORM improved their clarity in the rendered 3D SM model to enable more accurate measurement of their dimensions.

The structural complexity of the nuclear envelope (Agrawal and Lele 2019) was reflected in the features we observed when imaging lamin B1. The folds and tunnels observed along the lamina surface could be related to the nucleoplasmic reticulum (NR) (Malhas et al., 2011), a feature observed to reach deep into the nucleoplasm through accumulation with lamin proteins (Drozdz et al., 2017). While its cellular function is not fully understood, aberrations to NR morphology are associated with diseased states (Shoeman et al., 2001; Bussolati et al., 2008; Cornelison et al., 2019). Nuclear blebs observed protruding from the main nuclear lamina (Figure 3D) could be a consequence of altered chromatin compaction (Stephens et al., 2018), or early signs of apoptosis (Lindenboim et al., 2020). Cone-like structures protruding inward of the lamina with openings measured in the range of several hundred nanometers (Figure 4F) have been identified previously as nuclear invaginations with SMLM (Schoen et al., 2017) and may have roles with lamin associated chromatin domains or intranuclear transport (Legartová et al., 2014).

## Discussion

Here we demonstrate that the photophysical properties of the organic fluorophore Alx647 are sufficient to provide an hour of continuous high laser power imaging up to 8 µm deep into fixed cell samples, enabling multiplane astigmatic 3D *d*STORM of whole nuclear lamina. Fluorophore performance did not deteriorate appreciably indicating potential for even longer imaging time, thereby allowing for greater *z* ranges than the ∼8 µm achieved here. This raises the possibility that multiplane 3D *d*STORM using Alx647 can also be useful not only for structures with greater *z* dimensions but also for imaging cells expanded with expansion microscopy (ExM) protocols. The emerging combination of ExM with SMLM (Zwettler et al., 2020) to yield cumulative resolution gain for so called ultra-resolution (∼2–5 nm) has revealed unprecedented structural detail of biological samples previously only achievable with electron microscopies. Since ExM typically incurs a ∼4-fold lateral expansion (Chen et al., 2015; Tillberg et al., 2016), with some protocols and iterative strategies achieving 10-fold expansion (Chang et al., 2017; Truckenbrodt et al., 2019), sample volume in 3D is equivalently increased.

Implementing adaptive optics (AO) onto microscope setups have shown to increase the viable imaging depth in a sample and has enabled astigmatic SM imaging up to 90 µm in *z* into the sample (Mlodzianoski et al., 2018). AO is also capable of engineering more complex PSF shapes that distort over larger *z* ranges of up to 6 µm (Shechtman et al., 2015), enabling a much greater *z* range in a single slice for 3D SMLM, which, when applied to multiplane *d*STORM of whole cells, would substantially reduce the number of *z* slices required and thereby the total imaging time and the occurrence of localization errors and artefacts. Ultimately, total imaging time is dependent on the resilience of fluorophores during extended *d*STORM acquisitions, which may be extended with more robust organic dyes and more accommodating switching buffer compositions (Nahidiazar et al., 2016), or by utilizing sophisticated experimental set-ups such as flow-through labelling systems that continuously replenish the fluorophore population (Venkataramani et al., 2018). Given the efficiency of Alx647 demonstrated in this work, and the fact that we did not detect an upper limit for SM imaging performance after 1 h, there is good reason to expect that Alx647 could be used for 3D *d*STORM or ultra-resolution over longer acquisition periods (2–3 h) to capture larger expanded samples potentially up to ∼20 µm in *z*, or more with the addition of adaptive optics.

Aberrant nuclear membrane phenotypes have been associated with disease pathologies (Muchir and Worman 2004; Fichtman et al., 2019). Examination of whole nuclei morphology with the detail from super-resolution imaging could enhanced visualization of previously uncharacterized structural changes brought about by virally-induced differentiation (Li et al., 2021), genetic anomalies, or drug treatments (Rozario et al., 2021). The nuclear lamina, as imaged in this work via lamin B1, also provides a distinct outline of the nucleus. Fitting with the convex hull provides a quantifiable reference boundary, useful for characterizing intranuclear distributions of chromatin regions along with chromatin features such as histone modifications, as well as other nuclear components including transcription factors and replication and repair proteins (Whelan and Rothenberg 2021), for example, and sub-nuclear structures such as nucleoli (Rawlinson et al., 2018). This would provide information about the nuclear interior at the single-cell level and would establish a standardized distribution parameter when interrogating multiple cells. Achieving this would require two imaging channels, one for lamin and one for the target of interest, and as such would require another suitable fluorophore, that is spectrally distinct from Alx647 and has desirable photophysical performance for whole cell 3D *d*STORM matching Alx647. Intranuclear features can then be spatially defined as a function of the nuclear lamina. For example, in the case of T cell nuclei that were observed to be closely spherical, radial plot analysis (Crosetto and Bienko 2020; Girelli et al., 2020) is a viable quantification method for the distribution of T cell intranuclear components. Because *d*STORM affords a highly precise SM coordinate list, more sophisticated analysis methods can be employed to map individual coordinates or coordinate clusters of the intranuclear components (Xu et al., 2018; Su et al., 2020) with respect the nuclear lamina.

Characterization of nuclear architecture, both in the interior and membrane-bound, remains challenging. The application of 3D super-resolution techniques, as well as next-generation ultra-resolution and AO imaging methods provide excellent visualization of specifically labelled proteins and structures. The structural detail provided with *d*STORM images and the accompanying SM coordinate dataset can be analyzed with various levels depending on the features of interest. We have shown that the convex hull analysis can extract useful parameters such as surface area and volume of the whole nucleus. Besides the convex hull fit, *d*STORM data may be processed using other methods that quantify volume such as the Voronoi tessellation-based 3DClusterViSu (Andronov et al., 2018). Another approach is to fit the 3D coordinates with an alpha shape (Gardiner et al., 2018), similar to the convex hull that wraps around the outermost datapoints, the alpha shape method additionally retains concave features, such as nuclear membrane folds observed from 3D ViSP models. Furthermore, the alpha shape method can be used for fitting internal surfaces of the nuclear membrane. Efforts to adapt the alpha shape method to quantify 3D SM data of the nuclear lamina are ongoing. The use of super-resolution microscopy to directly visualize and precisely quantify specific nuclear features is emerging as a powerful tool for investigations of genetic disease (Xu et al., 2020), immune cell functionality (Carr et al., 2017; Ponjavic et al., 2018), and understanding how chromatin is organized *in situ* (Otterstrom et al., 2019; Xu and Liu 2021)*.* Achieving a complete nuclear atlas warrants further development in both the imaging technologies and analytical capabilities.

## Conclusion

Our assay extending SM PSF astigmatism for multiplane 3D *d*STORM of whole nuclear lamina demonstrates the capability of modern modular microscope setups and harnesses the superior performance of the Alx647 organic fluorophore for over 1 h in a simple photoswitching buffer. The rendered 3D images encompassed the entire nuclear dimensions while retaining the excellent subdiffraction resolution (∼20 nm in *x* and *y* and ∼50 nm in *z*) achieved with *d*STORM. Convex hull analysis allowed quantification of nuclear surface area and volume that revealed the sphericity of T cells by their SA:V values of ∼1. Observed 3D structures included membrane folds, blebs and invaginations that each may contribute or be consequence of specific nuclear phenotypes. We expect that imaging the nuclear lamina to determine the inner nuclear envelope boundary is ideal for quantifying spatial distributions of nuclear contents such as histone modifications which can readily be labelled and mapped using super-resolution methods.

## Data Availability

The datasets presented in this study can be found in online repositories. The names of the repository/repositories and accession number(s) can be found below: doi: 10.26180/19358699.
