# Supplementary material for: 3D Single Molecule Super-Resolution Microscopy of Whole Nuclear Lamina
Source: Front Chem. 2022 Apr 28;10:863610. doi: 10.3389/fchem.2022.863610 (PMC9096160; doi:10.3389/fchem.2022.863610)
Supplement: Supplementary file 2 [file DataSheet1.docx]

Supplementary Material

##
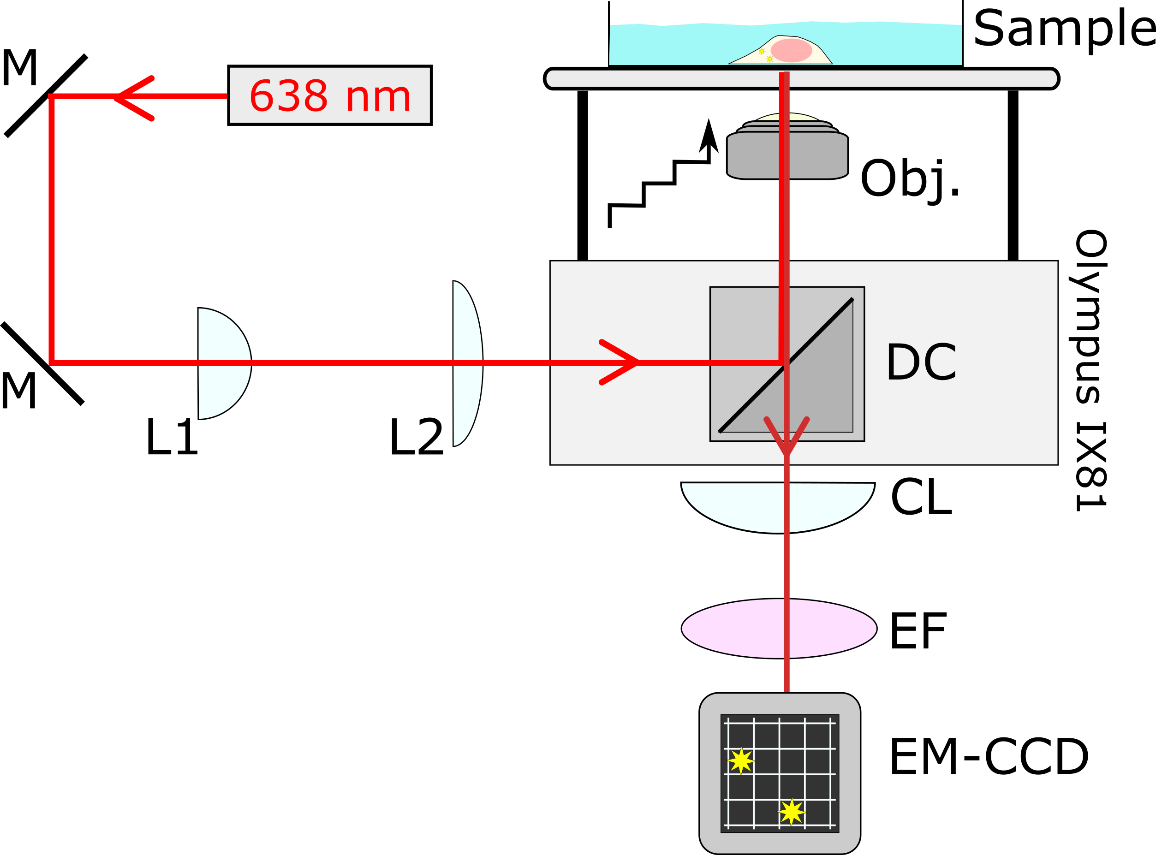
Supplementary Figures

**Figure S1.** Schematic of microscope setup for 3D multiplane *d*STORM. 638-nm laser diode excitation is directed with mirrors M and expanded using lenses L1 (f = 19 mm) and L2 (f = 100 mm) to fill the back of the objective in the Olympus IX81 microscope frame. The expanded laser is reflected onto the sample with the dichroic cube DC (Chroma zt532/640). For 3D multiplane *d*STORM, the objective is raised in a stepwise manner to capture multiple depths of the cell sample. Fluorescence is collected by the objective, directed through the DC and out the microscope frame through the cylindrical lens (f = 1000 mm), then through an emission filter bandpass EF (Chroma 700/75) before detection with the Andor iXon EM-CCD detector.

**
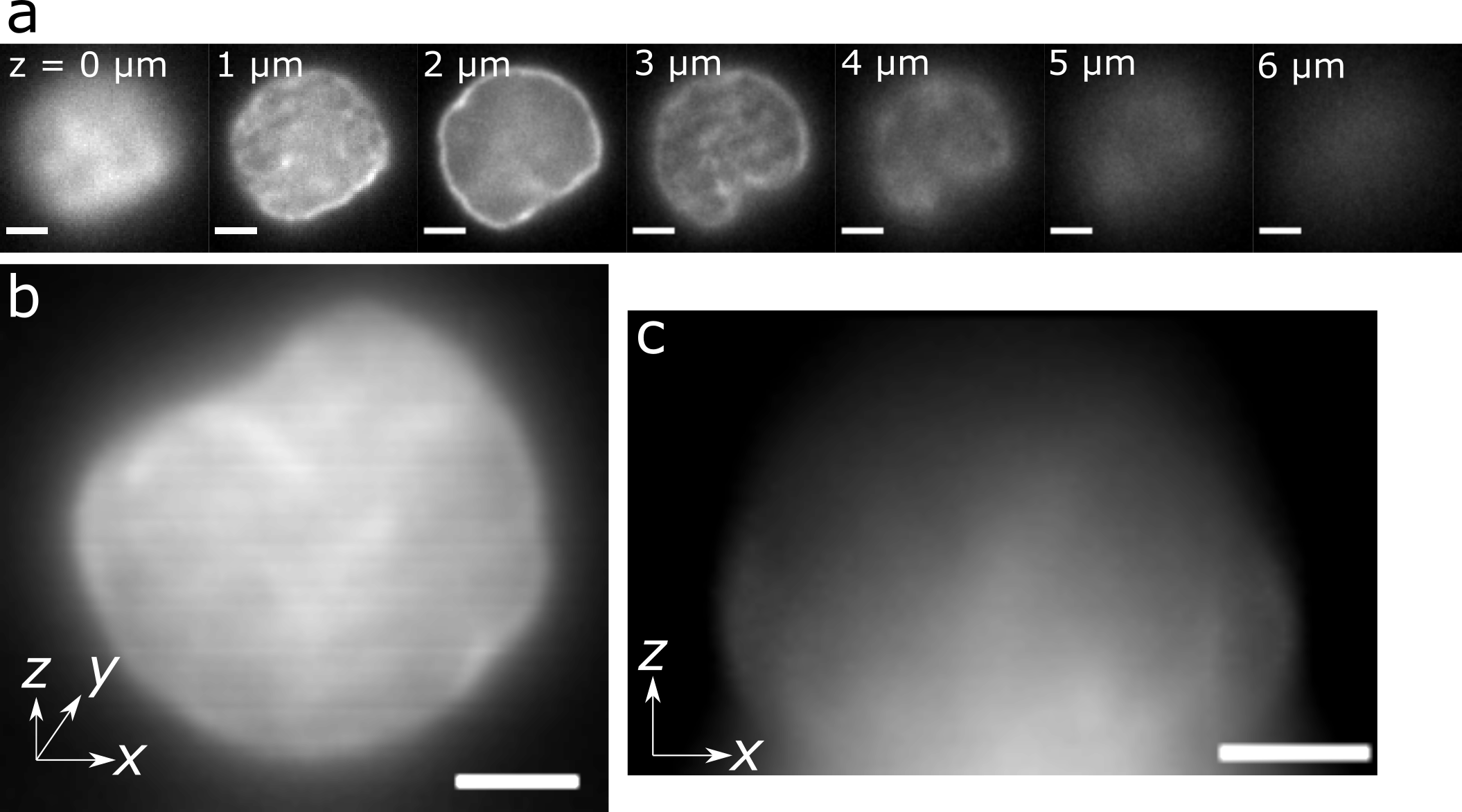
 Figure S2** **(a)** Diffraction limited widefield imaging of T cell nuclear lamina at each 1-µm *z* increment. **(b)** 3D visualization (tilted) of stacked widefield images captured every 100 nm in *z*. **(c)** Side on *xz* visualization of nucleus in (b) showing outline of lamina surface and decrease in average fluorescence signal toward the top of the cell compared to the base. Scale bars = 2 µm.


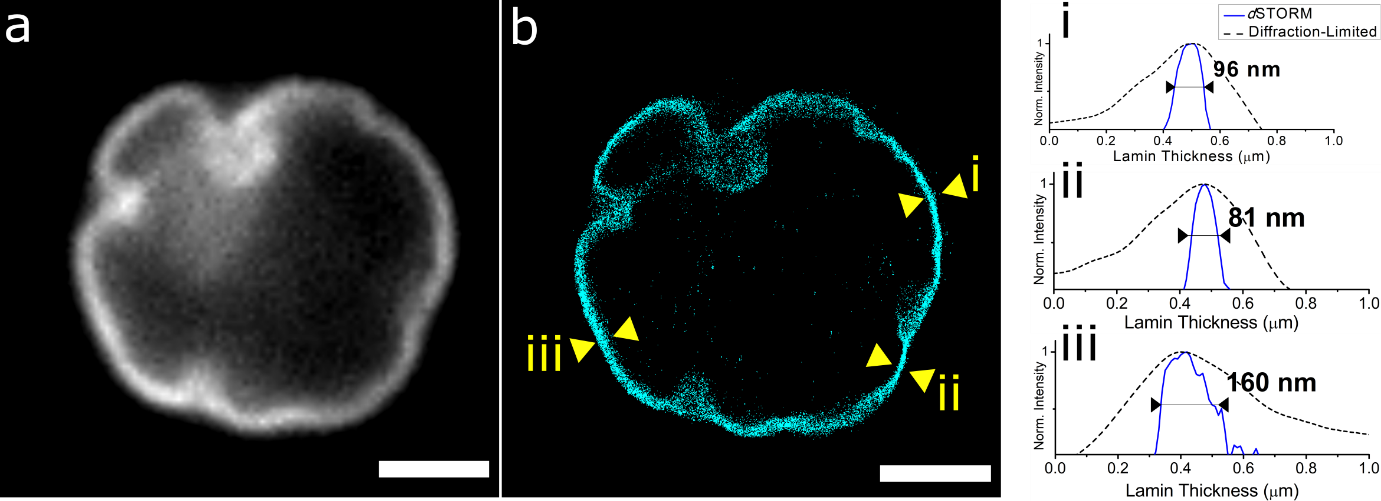


**Figure S3**. *d*STORM improves resolution of nuclear lamina periphery in T cells. Middle plane (*z* = ~ 3 µm) of a T cell nuclear lamina captured under widefield illumination **(a)** and using *d*STORM **(b)**. **(i, ii, iii)** Intensity cross-sections of nuclear lamina at points indicated in (b) (yellow marks) where the improved resolution of the *d*STORM image enabled subdiffraction measurements of lamina width. Scale bars = 2 µm.

**
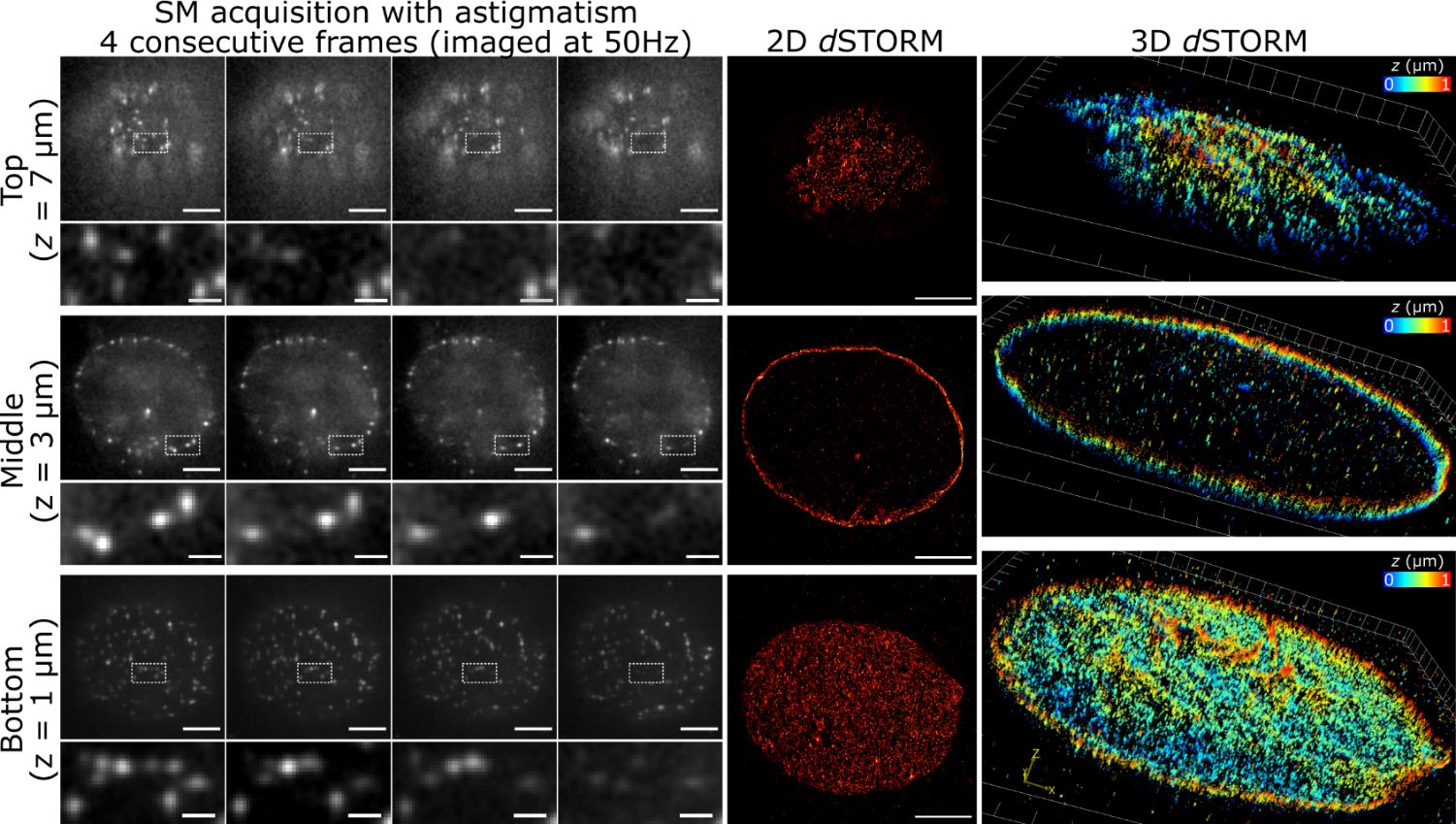
**

**Figure S4**. (left column) Raw data acquired for 3D *d*STORM showing lateral astigmatism in detected SM emissions, imaged at the bottom, middle and top of COS-7 nuclear lamina (labelled for lamin b1). (centre column) Rendered 2D *d*STORM images, from rapi*d*STORM, of each slice comprising 1 µm depth. (right column) Rendered 3D *d*STORM images, from ViSP, of each slice with SM coordinates colour coded for height up to 1 µm depth.

**
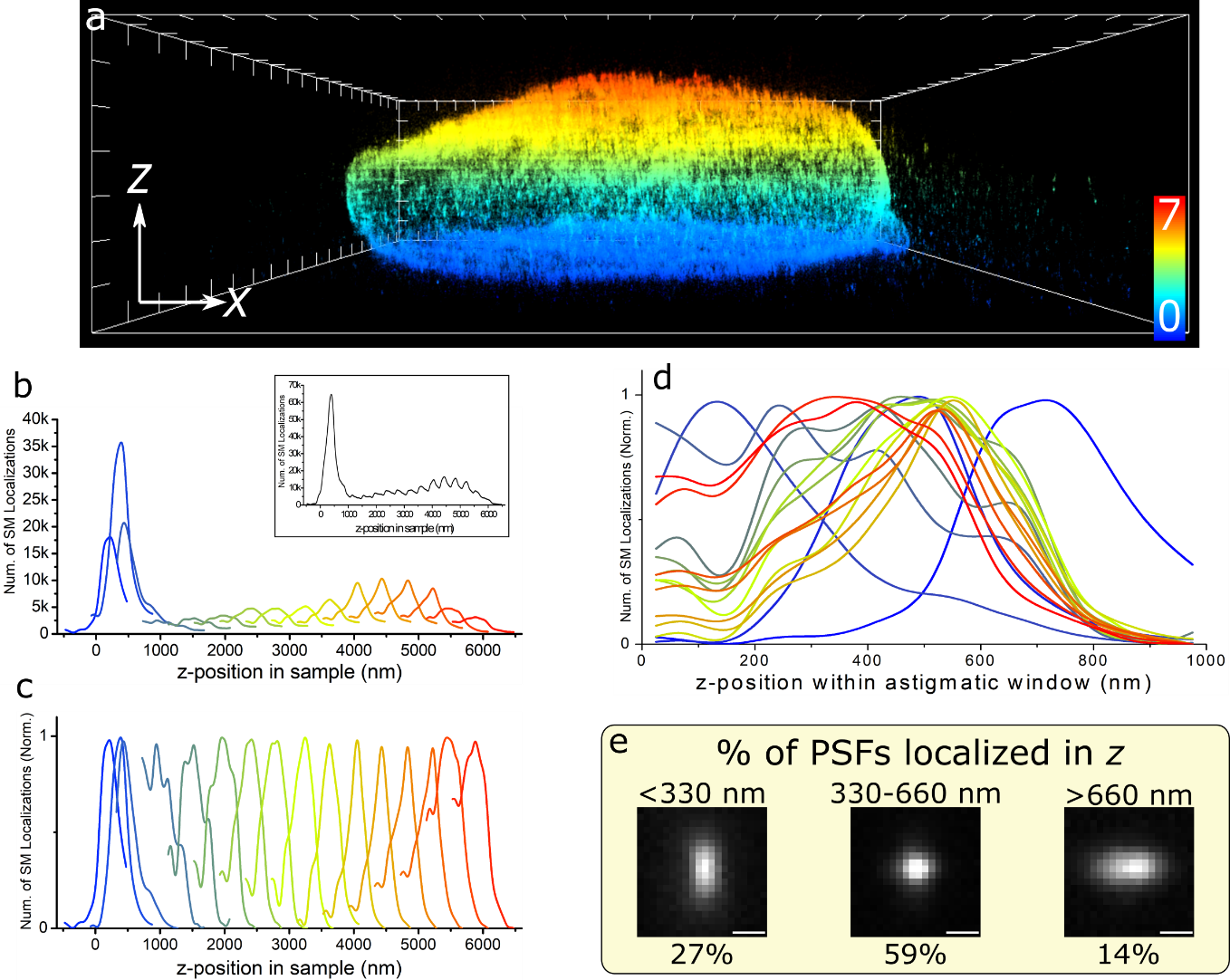
**

**Figure S5. Striping artefacts in *z* occur due to less efficient localization of highly distorted PSFs. (a)** Side on view (*xz*) of whole T cell nuclei imaged with 3D en bloc *d*STORM shows striping occurring along the *z*-axis. Grid ticks = 1 µm. **(b)** Number of localized SMs over the height of cell in (a) from each of the 16 overlapping *z*-steps (blue to red = bottom to top). Inset graph shows the compiled distribution of SM localizations. **(c)** Each plot in (b) normalized for number of SM localizations reveals a common distribution among z-steps where most localizations are from their middle section forming a distinct peak. **(d)** All normalized plots from (c) as a function of ­*z*-position within the 1 µm axial window. The plot on the far right in blue is the first ­*z*-step of the nucleus where the objective’s ideal imaging plane was just below the base on the lamina, resulting in most of the localizations occurring at the higher end (*z* >600 nm) of the astigmatic window. **(e)** Images are of calibrated PSF elongations occurring either below (left), in the middle (centre), or above (right) the objective imaging plane (as previously described). Scale bars = 500 nm. An approximate breakdown of the generalized PSF shapes used to form plots in (d). Less elongated PSFs in the middle section of the window (*z* = ~330 – 660 nm) are the most to be localized, followed by *y*-elongated PSFs (*z* < 330 nm) at the bottom end, then *x*-elongated PSFs at the top end (*z* > 660 nm).


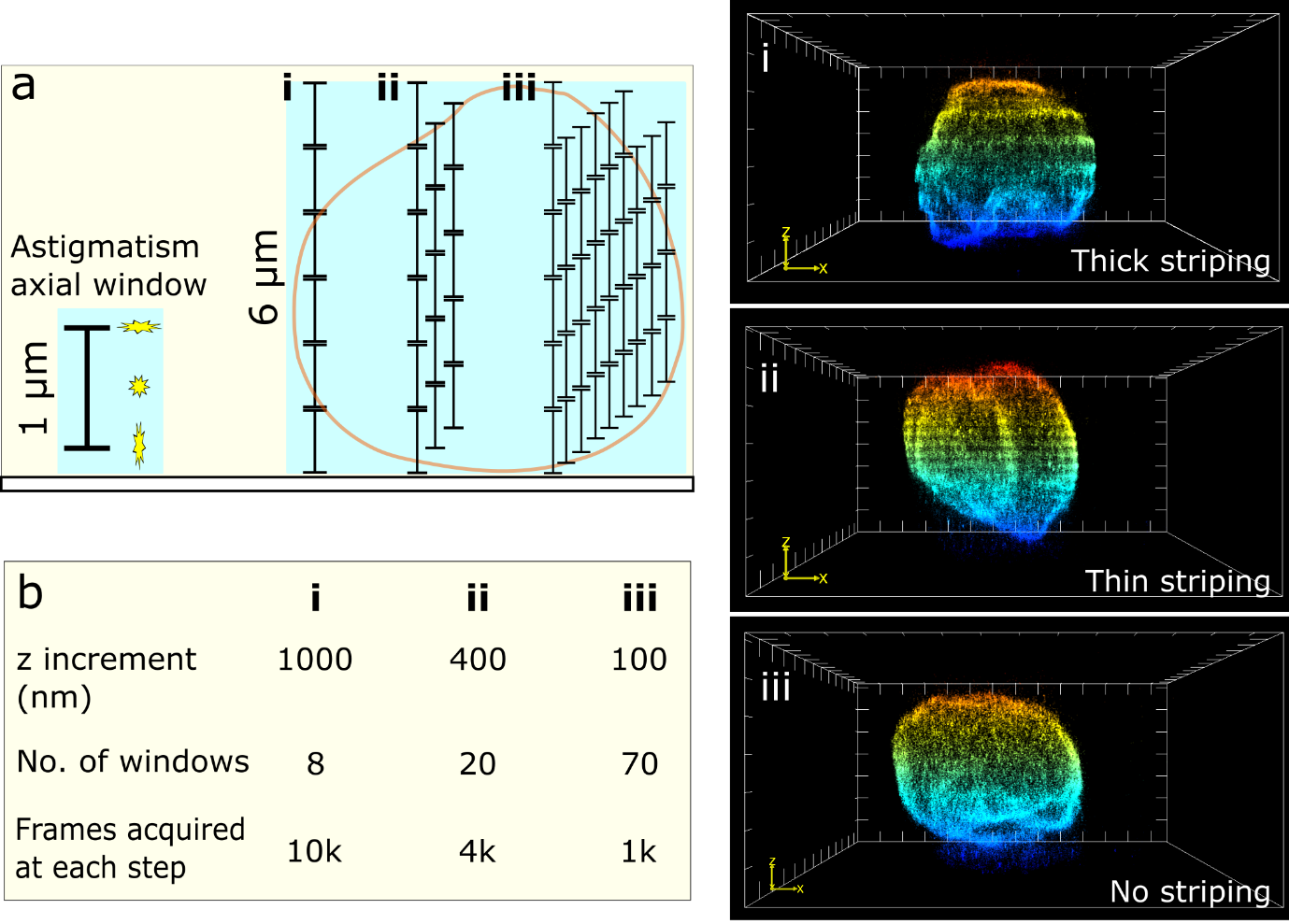


**Figure S6.** Effect of overlapping astigmatism *z* windows (slices) with multiplane 3D *d*STORM to image T cell nuclear lamina. **(a)** Schematic of imaging a 6 µm tall nucleus with different numbers of *z* slices and different extents of overlapping. **(b)** Imaging parameters used for each of the three conditions tested (i, ii, iii) and the resulting 3D models viewed from the side in the ViSP model with SM coordinates colour coded for height up to 6 µm from blue to red. With more axial windows, and thus more overlapping, less striping artefacts are observed in the resulting 3D model.
